# Supplementary material for: Are carbon emissions trading and green financial instruments synergistic? -Comprehensive quantitative research based on content analysis
Source: PLoS One. 2024 Mar 7;19(3):e0298601. doi: 10.1371/journal.pone.0298601 (PMC10919601; doi:10.1371/journal.pone.0298601)
Supplement: S1 Data — (ZIP) [file pone.0298601.s001.zip › Data/地级市Z_F工作报告.zip!/║╙─╧/░▓╤⌠╩╨/└┤╫╘╣1⁄2╓┌║┼í╛┬φ┐╦╩2╛▌═°í┐_2019.docx]

各位代表：

    现在，我代表市人民政府，向大会报告工作，请予审议，并请各位政协委员和其他列席人士提出意见。

    一、2018年工作回顾

    过去一年，面对严峻复杂的国际国内形势和艰巨繁重的改革发展任务，在省委、省政府和市委的正确领导下，全市上下以习近平新时代中国特色社会主义思想为指导，全面落实党的十九大精神和习近平总书记视察指导河南时的重要讲话精神，坚持稳中求进工作总基调，以新发展理念为引领，以高质量发展为方向，以供给侧结构性改革为主线，深入开展三大攻坚战，统筹推进“四个着力”、打好“四张牌”的安阳实践，经济社会发展总体平稳、稳中有进，实现“一个重返、六个重大”、建设新时代区域性中心强市、助力中原更加出彩迈出新步伐。

    一年来，我们主要做了以下工作：

    （一）抓住关键环节，稳定经济增长。妥善处理调结构与稳增长、治污染与稳运行的关系，科学施策，精准发力，全市经济运行保持在合理区间。初步核算，全市生产总值2393亿元，增长6.7%。

    着力稳需求。实施“6115”扩大有效投资计划，激活民间投资，稳定有效投资，高铁站东广场、郑济高铁安阳段等加快建设，西北绕城高速公路、新东北外环建成通车，人民大道拓宽改造圆满完成，洹河东湖闸、西湖闸等一批水利工程竣工投用，省、市重点项目年度投资目标超额完成。推进健康、体育、文化、旅游等领域基础设施建设，培育消费热点，优化消费环境，提振消费信心，社会消费品零售总额增长9.4%。对外贸易逆势前行，完成出口33.3亿元，增长38.1%。

    着力降成本。认真落实国家和省减税降费系列政策措施。降低企业社会保险费率，减轻企业负担1.7亿元。推进电力直接交易，清理规范转供电，降低一般工商业用电成本6.9亿元。

    着力保要素。实施“双百融资对接行动”，开展“金融服务中小企业进园区”活动，促进企业从银行、保险、证券、基金等多渠道融资，新增贷款163亿元，直接融资150亿元。落实失业保险稳岗补贴1131.6万元，帮助企业稳定就业岗位4.5万个。强化就业服务和技能培训，着力提升劳动力素质，有效缓解企业招工难问题。推进“三地一矿”综合改革，重点工程建设和企业生产用地需求得到有效保障。

    （二）推进转型升级，提高发展质量。持续深化供给侧结构性改革，建立完善“1+6+16”转型攻坚体系，着力提升供给质量效率。四大千亿级主导产业增加值增长12.8%，高于规模以上工业增加值增速7.1个百分点，主营业务收入超50亿元企业达到8家。全市一般公共预算收入154.1亿元，增长18.9%；税收占比76%，比上年提高4.3个百分点。

    传统产业延链提质。着力推进链条延伸、产品提质、技术提升、装备提档、管理提效，传统产业加快向产业链和价值链中高端攀升。实施绿色化、智能化和技术改造，一批绿色工厂、智能车间建成投用。强化环保、安全、能耗、技术、质量等标准约束，一企一策推进23个行业深度治理、提标改造，骨干企业适应环保常态化管控和市场竞争能力持续增强。产品结构持续优化，安钢高强度板、锅炉和压力容器板市场占有率全国第一，汽车大梁钢销量行业领先；凤宝热扩车桥、晨盛装配式钢结构、复星合力高延性冷轧钢筋等项目加快建设。煤化工循环经济产业链基本形成，四大焦化企业非焦产品产值比重超过60%。食品医药、纺织服装等产业集群化、品牌化发展水平进一步提升。

    新兴产业集聚壮大。着力抓机遇、上项目、建基地，强化龙头企业引进，战略性新兴产业加快发展。新能源汽车及零部件产业实现新突破，德力新能源商用车成功下线，比亚迪安阳基地、林州汽车零部件产业园建设稳步推进。通用航空产业取得新进展，通航制造中心一期主体竣工，陕西金宇、贯辰万基、宿迁飞鹰等一批通航项目顺利入驻。新材料产业迈出新步伐，光远电子玻纤、电子布三期和致远、诚雨覆铜板等项目建成投用。智能制造产业呈现新活力，哈工大、奥特瑞等一批机器人项目签约落地。

    现代服务业提速增效。加快文化旅游融合发展，推进重点景区道路连通、门票降价、服务提质，接待国内外游客、旅游总收入分别增长12.6%和16.1%。积极培育现代金融业，金融服务实体经济能力增强。大力发展现代物流，成功入选陆港型国家物流枢纽承载城市，万庄公铁物流园被确定为省多式联运示范工程。邮政、快递业务量分别增长22.5%和20%。电子商务交易总额282亿元。服务业对经济增长贡献率达到55.8%，成为拉动经济增长主动力。

    （三）聚力三大攻坚，弥补发展短板。坚持把打好三大攻坚战作为各项工作的重中之重，系统谋划，精准施策，务实推进。

    有效防范重大风险。强化组织协调、风险预警，完善债权人委员会制度，有效防范企业担保链、资金链风险。切实防范金融风险，深入开展非法集资陈案化解、大案攻坚、“半拉子”工程治理，一批问题企业、问题项目、问题楼盘、问题工程得到有效处置。认真落实“八个一批”措施，政府债务风险总体可控。非洲猪瘟防控取得阶段性成果。统筹做好社会稳定、公共安全、自然灾害等领域风险防范工作。

    扎实开展精准脱贫。坚持精准方略，聚焦脱贫质量，实施产业扶贫、就业创业扶贫等重大专项，积极推进结对帮扶，巩固易地搬迁扶贫成效，持续改善重点贫困地区和特殊贫困群体发展条件。年度减贫目标全面完成，12916人摆脱贫困，38个贫困村脱贫退出，内黄县达到脱贫摘帽条件，打赢脱贫攻坚战三年行动实现良好开局。

    强力推进污染防治。深入落实全国、全省生态环境保护大会精神，制定实施污染防治三年行动计划，推进科学治污、依法治污、精准治污、全民治污。突出重点区域、重点行业、重点时段，标本兼治，科学管控，PM10、PM2.5年日均浓度完成年度目标，降幅均居全省第四位，全市优良天数同比增加，实现“两降一增”，综合指数改善率居全省第一位。全面落实河长制，实施“堵污口、清污泥、治污水、净水质”专项清河行动，开展河道采砂综合治理，黑臭水体治理和省、市流域重点工程建设基本完成，河流水质明显改善。推进国土绿化，营造林19.3万亩，森林覆盖率达到31.9%。重度污染耕地种植结构调整等试点工作稳步实施。露天矿山综合整治取得初步成效。

    （四）统筹城乡建设，提升发展品质。以文明城市创建为引领，实施城市建设提质工程，加快推进以人为核心的新型城镇化，实施乡村振兴战略，城乡发展更加协调。常住人口城镇化率提高1.8个百分点左右。

    推进城市建设提质。做好“四篇文章”，强化“四治”要求，推动城市内涵式发展，顺利通过国家文明城市提名城市年度验收和国家卫生城市复审，城市综合承载能力和城市品质进一步提升。中心城区新增绿地1.14万亩，迎宾公园、CBD公园等8个大型公园建成开放，人均公园绿地面积达到11.86平方米。引岳入安二期、示范区中轴线生态水系等项目加快推进，第六水厂和第四水厂二期通水运行。古城保护整治复兴工程启动实施，仓巷街恢复历史风貌。开展城市“双修”，实施文峰大道中段、紫薇大道等示范街区整治工程，改造提升一批老旧小区，拆除违建162.8万平方米，新增集中供暖面积327万平方米。强化城市管理，推进生活垃圾分类，市政道路整修、背街小巷整治、“两场一所”建设按期完成，城市的一些痛点、堵点、热点问题得到有效缓解。

    扎实推进乡村振兴。制订全市乡村振兴战略系列规划和专项行动计划，突出重点，分类施策。坚持以农业结构调整为主线，促进农业强。加快“四优四化”建设，粮食总产375万吨，新增高标准粮田16.3万亩，调减玉米种植面积28.6万亩，优质专用小麦、优质花生、设施瓜菜种植面积进一步扩大。农业产业集群收入突破360亿元。坚持以提高农民收入为核心，促进农民富。改善农民生活条件，增强农民保障能力，全市农村居民人均可支配收入增长8.3%，高于城镇0.8个百分点。坚持以改善农村人居环境为重点，促进农村美。89个村完成综合整治，2117个村实现生活垃圾有效治理，专项清理“大棚房”，完成农村危房改造2493户，新建改建标准化村卫生室171个、综合文化中心1115个，建成“四好农村路”302公里。改造农村户用厕所36.2万户，农村“厕所革命”工作受到省政府表扬。全省实施乡村振兴战略现场会在我市召开。

    （五）坚持改革开放，增强发展活力。以庆祝改革开放40周年为契机，深化改革强动能，扩大开放增优势，聚力创新促转型，发展的动力和活力不断释放。

    坚定不移推进改革。市级政府机构改革基本完成。以“放管服”改革为带动，优化营商环境，推进审批服务便民化，企业和群众关注的一批事项实现“最多跑一次”，重大项目审批时间又压减一半，企业注册登记审批时间缩短至4天。全年新增市场主体1.03万家。召开促进非公经济健康发展大会，制定实施“1+8”支持政策。国企改革继续走在全省前列。深化公立医院改革，市人民医院成为国家级现代医院管理制度试点。财税、价格、土地、环保、投融资、综合执法等领域改革稳步推进。

    持续提升开放水平。充分发挥开放招商一举多效的综合带动作用，以开放促改革、促转型、促发展。围绕主导产业、优势产业，紧盯长三角、珠三角、京津冀重点区域和国际国内知名企业，实施大员招商、小分队招商、以商招商，招商引资的专业性、精准性、有效性进一步增强。成功举办第十届航空运动文化旅游节，积极参加中国国际进口博览会、河南国际投资贸易洽谈会等重大招商活动。“三新”产业园、安井食品华北生产基地、迪尚服装等一批项目落户安阳。与中建材、华润、大唐等央企合作进一步深化。全年引进省外资金720亿元，实际利用外资5.27亿美元。

    不断增强创新活力。围绕培育引进创新引领型企业、平台、人才、机构，汇聚创新资源，增加研发投入，研究与试验发展经费占全市生产总值比重提高0.2个百分点。新增国家级高新技术企业16家。新挂牌院士工作站2家。新增省级工程技术中心4家、省级重点实验室1家。省级以上博士后创新实践基地达到14家。实施“慧聚安阳”行动计划，积极引进高层次人才和创新创业团队。红旗渠科技产业园成功创建国家小微企业创业创新示范基地。翔宇医疗获得“河南省省长质量奖”。顺利通过国家知识产权示范市复核验收。

    （六）加强社会建设，增进人民福祉。坚持以人民为中心的发展思想，着力解决关系群众切身利益问题，全年财政民生支出274.3亿元，省定市定民生实事较好完成。

    社会就业保持稳定。城镇新增就业8.45万人，新增农村劳动力转移就业3.2万人。返乡下乡创业人数达到8万人，累计带动就业46.7万人。城镇登记失业率控制在3%以内。

    社会保障不断完善。养老金实现十四连增。城乡居民基本医疗保险、大病保险、困难群众大病补充医疗保险实现一站式即时结算，基层医疗卫生机构一般诊疗费纳入城乡居民基本医疗保险支付范围。城乡低保年人均标准分别增加480元和350元。城乡特困人员基本生活供养标准进一步提高。新开工建设棚改安置房2.3万套。

    社会事业全面发展。优先发展教育事业。新建改扩建幼儿园56所。改扩建农村寄宿制学校16所。新增学位5.2万个。义务教育超大班额问题基本解决。167个“全面改薄”项目圆满完成。市一中新校区、市实验幼儿园新园加快建设。着力增加优质医疗资源供给，市妇幼保健院晋升三级医院，全省区域中医儿科诊疗中心落户我市。医疗机构新增床位4500张。完成消化道肿瘤筛查24万人、适龄妇女两癌筛查5.7万人。家庭医生签约服务实现贫困人口全覆盖。持续加强文物保护，殷墟遗址安全整治受到国家、省相关部门肯定，殷墟国家考古遗址公园规划顺利获批，成功举办殷墟科学发掘90周年纪念大会。基层公共文化服务体系不断完善。全民健身运动广泛开展，竞技体育水平不断提升。

    社会治理持续加强。深入开展扫黑除恶专项斗争。依法做好信访工作。村（社区）“两委”换届圆满完成。强化困难群体法律援助。加强食品药品安全专项治理。安全生产实现“三下降一杜绝”。社会大局保持和谐稳定。

    过去一年，我们自觉接受人大监督、政协民主监督和社会监督，认真办理人大代表建议和政协提案。积极推进依法行政，强化审计监督，持续纠正“四风”，以敬畏之心抓好中央巡视反馈问题整改，政府自身建设取得新进步。加强国防教育和国防后备力量及人民防空建设，军民融合深度发展，“双拥”共建持续深化，海军安阳舰成功入列，驻安人民解放军、武警部队、预备役人员、民兵为地方发展作出新贡献。扎实推进民族工作，依法规范宗教事务，宗教工作法治化水平进一步提高。妇女儿童、老龄、残疾人、红十字、慈善等事业健康发展。老区建设不断加强。统计、史志、档案、气象、地震、外事侨务、发展研究、援疆等工作取得新进展。

    这些成绩的取得，是习近平新时代中国特色社会主义思想科学指导的结果，是省委、省政府和市委坚强领导的结果，是市人大、市政协大力支持的结果，是全市干部群众凝心聚力、拼搏奋斗的结果。在此，我代表市人民政府，向全市各族人民，向驻安人民解放军指战员、武警部队官兵、公安干警，向各民主党派、工商联、各人民团体、新闻媒体及社会各界人士，致以崇高的敬意！向关心支持安阳发展的港澳台同胞、海外侨胞和国际友人，表示衷心的感谢！

    各位代表！

    前进路上无坦途。总结成绩的同时，我们也清醒认识到，经济社会发展还面临不少困难和问题。一是下行压力持续增大。去年我市经济运行前高后低，增速逐季回落，部分主要经济指标出现较大波动。今年受宏观环境不确定性因素增多影响，矛盾和问题碰头叠加，经济运行稳中有变、变中有忧，稳增长难度加大。二是结构矛盾依然突出。产业结构不合理，冶金建材、煤化工等行业占比过高，新产业、新业态、新模式规模较小，新旧动能转换较慢。特别是随着资源环境和市场约束趋紧，短期内我市稳增长与治污染、保态势与优结构的统筹更为困难。三是风险隐患不容忽视。规模以上工业企业资产负债率偏高，一些企业债务链、担保链交织，存在连锁违约风险。房地产市场走势存在不确定性。四是实体经济困难较多。随着市场需求增长放缓，资金、能源原材料、人工、用地成本较高，企业盈利空间受限，部分企业经营困难加重，担忧情绪、避险情绪、观望情绪上升，信心不足。五是民生领域还有短板。城乡居民持续增收制约因素增多，贫困群众生活改善任务较重，生态环境形势依然严峻，教育、医疗、养老等公共服务与人民日益增长的美好生活需要仍有较大差距。同时，政府工作存在不足，有些改革和政策措施落实不到位，一些干部怕问责、不担责，存在推拖庸懒、不作为慢作为等现象，一些领域不正之风和腐败问题依然时有发生。对此，我们一定拿出有效办法，认真加以解决，尽心竭力把各项工作做好！

    二、2019年工作安排

    今年是新中国成立70周年，是全面建成小康社会的关键一年，也是我市加快新旧动能转换、实现高质量发展的重要一年，做好今年的工作意义重大。

    今年政府工作的总体要求是：坚持以习近平新时代中国特色社会主义思想为指导，全面贯彻党的十九大和十九届二中、三中全会精神及中央经济工作会议精神，持续落实习近平总书记视察指导河南时的重要讲话精神，深入落实省委十届八次全会和市委十一届九次全会部署，统筹推进“五位一体”总体布局，协调推进“四个全面”战略布局，坚持稳中求进工作总基调，坚持新发展理念，坚持高质量发展根本要求，坚持转型发展主攻方向，坚持供给侧结构性改革，贯彻巩固、增强、提升、畅通的方针，统筹推进稳增长、促改革、调结构、惠民生、防风险、保稳定工作，抓重点，补短板，强弱项，保持经济平稳健康发展和社会大局稳定，增强人民群众获得感、幸福感、安全感，为全面建成小康社会收官打下决定性基础，在实现“一个重返、六个重大”目标任务、建设新时代区域性中心强市征程中迈出更大步伐，以优异成绩庆祝中华人民共和国成立70周年。

    经济社会发展主要预期目标是：经济运行保持在合理区间，生产总值增长7%—7.5%，全员劳动生产率增长7%左右，一般公共预算收入增长8%左右，其中税收占比高于全省平均水平，规模以上工业增加值增长7%左右，服务业增加值占生产总值比重达到45%以上，固定资产投资增长8%左右，社会消费品零售总额增长9%左右，居民消费价格涨幅控制在3%左右，经济结构进一步优化，高质量发展取得积极进展。创新发展取得新成果，研究与试验发展经费投入强度达到1.2%，四大千亿级主导产业增加值增长10%以上，高新技术产业、战略性新兴产业增加值占规模以上工业比重明显提高。协调发展取得新成效，常住人口城镇化率提高1.5个百分点以上，九年义务教育巩固率达到96.5%，医疗卫生、文化、体育等社会事业全面进步。绿色发展取得新提升，单位生产总值能耗、PM10、PM2.5、空气优良天数等节能减排、环境保护指标完成省下达任务。开放发展取得新突破，进出口保持平稳增长，实际利用外资、引进省外资金完成省定目标。共享发展取得新进步，居民人均可支配收入增长7.5%左右，城镇新增就业6.5万人，城镇登记失业率控制在4%以内。以上目标，更加凸显了新发展理念和高质量发展要求，有利于全市上下进一步集中力量、突出重点，更好推进高质量发展。

    贯彻总体要求，实现预期目标，必须把思想和行动统一到中央、省委对形势的分析判断上来，统一到市委对下步工作的安排部署上来，在形与势的统一中审视全局，在危与机的转换中抢抓机遇，在供与需的重构中深化改革，在稳与进的把握中迎难而上，推动全市经济社会不断向前发展。具体工作中，要努力把握好“五个必须”。一是必须坚持稳中求进，把发展稳住。发展是第一要务，是解决一切问题的关键。要正确处理稳增长与调结构提质量的关系、与抓改革强动力的关系、与防风险降杠杆的关系、与保就业惠民生的关系，统筹抓好各领域工作，优化供给质量，稳定发展预期，提振发展信心，确保经济运行在合理区间。二是必须坚持底线思维，把风险管住。当前各类风险隐患叠加，一旦哪个方面引爆，极易产生连锁反应。要紧盯经济发展、社会稳定、自然灾害、公共事件、安全生产等领域，不忽视一个风险，不放过一个隐患，守土负责、守土尽责，抓住要害、找准原因，完善机制、有效处理，坚决守住不发生系统性区域性风险的底线。三是必须坚持聚焦实体，把转型扭住。推动经济高质量发展，重点是推动产业结构转型升级，做实做强做优实体经济，构建现代化经济体系。要牢牢把握高质量发展根本方向，推动资源要素向实体经济集聚、政策措施向实体经济倾斜、工作力量向实体经济加强，优化发展环境，加快经济结构转型、发展方式转变、增长动能转换。四是必须坚持解放思想，把机遇抓住。解放思想是发展的总开关，没有思想上的破冰，就没有行动上的突围。要在回看走过的路中增添信心，在比较别人的路中拉高标杆，在远眺前行的路中创造机遇。积极抢抓当前中央稳增长、补短板、强弱项的重大机遇，顺势而为，乘势而上，有力有序有效推进基础设施建设。要充分发挥我市区位、产业、资源等比较优势，全面提高对外开放水平，重塑竞争优势，坚决把接下来的路走好，在新一轮发展中争取主动、赢得先机。五是必须坚持发展为民，把民生兜住。坚持把关系群众切身利益的关键小事作为政府工作的头等大事，持续加大民生投入，增强社会保障能力，稳定扩大社会就业，不断提高居民收入，切实办好民生实事，让改革发展成果更多更公平地惠及全市人民。

    今年重点抓好以下七个方面工作：

    （一）稳中求进，巩固发展态势。贯彻落实“六稳”工作部署，把“稳”和“进”有机统一起来，以稳求进，以进促稳，确保经济平稳增长。

    稳定工业经济。工业是实体经济的主体，工业稳则经济稳。持续加强工业经济运行分析研判，着力抓好能源、运输、资金等要素保障，落实减税、物流、金融、电力、社保等降成本政策，做好产销、产需、产运对接。深化增品种、提品质、创品牌行动，提高产品竞争力和市场占有率。实行环保绿色调度，落实差别化管控政策，支持绿色环保工业企业优先生产。优化企业服务，开展政策落实进万企活动，严格落实企业反映问题市长交办制度，一企一策帮助企业解决实际问题。

    扩大有效投资。实施补短板稳投资专项行动，推进500个以上重大项目建设，确保全社会固定资产投资稳定增长。建立政府专项债券项目安排协调机制、政银企对接重大项目清单。积极争取中央政策性资金和专项债券支持，用足开发性、政策性和商业性金融资金，用好用活政府与社会资本合作政策，充分调动民间投资，提升资金保障能力。建立完善项目前期推进机制，全力保障项目按期动工。

    激发消费潜力。适应消费升级新形势，满足人民群众对中高端服务消费和物质产品消费的需求，多渠道扩大优质服务供给。培育消费热点，扩大移动互联网、物联网等信息消费，推广新能源汽车、智能化家电等绿色消费，推进旅游休闲和文化体育消费，鼓励健康养老消费。放宽服务消费领域市场准入。开展家政服务标准化试点。落实个人所得税专项附加扣除政策。强化消费市场监管，加大守信激励和失信惩戒力度，改善消费环境。稳定房地产市场，合理引导社会预期，逐步建立多主体供给、多渠道保障、租购并举的住房制度。

    （二）调转并重，提升质量效益。全面落实“八字方针”，持续深化供给侧结构性改革，突出培育壮大四大千亿级主导产业，促进经济高质量发展。

    推进制造业转型升级。以制造业为根基，强力推动传统产业转型攻坚、战略性新兴产业发展壮大。加快钢铁行业整合重组，进一步优化产业布局，促进生产绿色化、装备大型化、企业智能化、钢材精品化，加快实现“三减三升”。推动焦化行业延伸产业链条，大力发展精细化工，持续提升非焦产品比重，坚决淘汰落后产能。继续推进“三大改造”，突出抓好绿色工厂创建、绿色园区建设，加快产品创新、工艺创新、管理创新、品牌提升。积极推进新能源汽车及零部件、智能制造、电子新材料等产业发展。支持德力新能源商用车达产，谋划争取新能源整车生产企业在安阳布局新的生产基地。深化同比亚迪合作，加快汽车零部件、智能终端、轨道交通设备制造安阳基地建设。加强与中建材合作，建设全国重要的药玻生产基地。以光远、诚雨、致远等龙头企业为引领，推动电子布、覆铜板等产品扩大规模、延伸链条、打造品牌，建设国内重要的电子新材料产业基地。推进通航制造产业园、智能机器人小镇等项目建设。

    加快服务业扩容提质。以文化旅游、现代金融、现代物流、健康养老为重点，促进服务业规模壮大、结构优化、质量提升。推动文化旅游产业加快发展，开工建设殷墟国家考古遗址公园，加快曹操高陵保护展示工程、大运河文化带展示提升工程、市旅游集散中心等项目建设，完善红旗渠、太行大峡谷等重点旅游景区配套服务功能，设计精品线路，加强景区互动，打造特色品牌，不断提升安阳旅游吸引力、影响力、竞争力。健全金融体系，丰富金融产品，改善金融生态，开展重点领域金融服务专项行动，提高金融服务实体经济能力，引进一批金融机构。加快建设陆港型国家物流枢纽承载城市。发展大宗商品物流、冷链物流、快递物流、电商物流，积极引进物流集成商，完善物流多式联运体系，提升物流配套能力，开工建设安西物流园，推进万庄公铁物流园、林州大运汽贸物流园等项目建设。创新医养结合模式，发展健康养老产业。发展数字经济、共享经济，促进互联网与实体经济融合发展。

    增强创新动力活力。聚焦“四个一批”行动，实施创新龙头企业提升引领工程、高新技术企业倍增工程、“科技小巨人”企业培育工程、大中型工业企业市级以上研发机构全覆盖工程，推动以技术创新为核心的全面创新。坚持以新技术应用和关键核心技术突破为牵引，引导企业加大研发投入，支持企业、高校、科研院所组建产业技术创新战略联盟及新型研发机构，共享大型科研仪器设备，提高产学研用水平。加强企业知识产权保护。强化开放式创新，落实人才政策待遇，促进高端研发机构、人才及创新成果落地安阳。

    提升载体发展水平。完善产业集聚区规划，健全信息、金融、检验检测、技术研发等新型服务平台，增强承载能力，提升创新发展水平，推进产业集聚区二次创业。开展产业集聚区、专业园区区域综合评价和企业分类评价，推行资源要素差别化配置，提升服务效率，加快项目落地，推动“龙头+配套”集群发展、“基地+基金”集聚发展、“存量+增量”同步发展。

    （三）聚焦聚力，打好三大攻坚战。紧盯全面建成小康社会短板，针对突出问题，打好重点战役，以更大力度推动三大攻坚战取得新成效。

    防范化解重大风险。时刻牢记习近平总书记“增强忧患意识、防范风险挑战要一以贯之”的要求，树牢底线思维，下好先手棋，打好主动仗。全面加强金融监管，支持银行业金融机构处置不良资产，着力防范区域金融风险。积极推进市场化债转股，引导企业加大股权融资力度，降低企业经营风险。高度重视企业担保链风险防范处置，“一企一策”隔离风险、阻断链条，防止传导蔓延。全力做好非法集资陈案化解，防范和打击新型非法集资，最大限度维护群众合法权益。依法规范政府举债行为，确保政府债务指标控制在合理区间。压实安全生产责任，全面推行双重预防体系建设，健全应急管理体系，坚决防范遏制各类事故发生。增强防灾减灾救灾能力，做好突发公共事件预防处置。

    扎实推进精准脱贫。突出产业和就业扶贫，大力发展农副产品深加工、乡村旅游、高效种养、服装加工等特色产业，更好吸纳贫困劳动力就业，促进贫困人口持续增收、稳定脱贫。发挥社会保障兜底作用，解决好贫困老年人、重度残疾人、重病患者等特殊贫困群体脱贫问题。统筹贫困村和非贫困村，聚焦“两不愁三保障”，加强农村基础设施建设，提升公共服务质量。建立脱贫正向激励和稳定脱贫长效机制，巩固提升滑县、内黄县脱贫成果，加强易地扶贫搬迁后续帮扶。强化市县主体责任、部门协调责任、乡村包干责任、驻村帮扶责任，确保再减贫2万人、33个贫困村脱贫退出。

    全面加强污染防治。坚持把污染防治作为推动高质量发展的关键之举、改善生态禀赋的长远之策、重塑安阳形象的必由之路、提升幸福指数的民生之要，扭住不放、全力攻坚，确保环境质量只能变好、不能变坏。聚焦打赢蓝天、碧水、净土保卫战，实施“四大行动”，优化“四个结构”。持续强化“六控”措施，继续抓好企业深度治理和无组织排放治理，实施生产全过程、厂区全覆盖、工艺全链条治理监控。综合运用差别电价、差别管控、规划、土地等政策措施，加快建成区企业退城进园、整合升级。强化能耗“双控”，加强散煤治理，大力推进清洁取暖“双替代”。优化西部运输结构，深入推进柴油重卡专项治理，探索建立城市绿色货运配送体系。科学精准做好重污染天气应急管控。强化“河长制”落实。抓好洹河、万金渠等水系清河行动。全域治理黑臭水体。继续开展河湖采砂专项整治。加强饮用水水源地保护。实施土壤污染防治三年行动计划，强化农业面源污染防治，加快建设静脉产业园，逐步改善土壤环境质量。

    （四）蓄能增势，深化改革开放。坚持把改革开放作为推动经济发展的动力引擎，全面落实各项改革举措，全方位扩大对外开放。

    推进重点领域改革。完成市县两级政府机构改革。持续深化“放管服”改革，继续压减一般性企业投资项目全流程审批时限，深度清理规范各类行政审批中介服务事项，清理规范涉企收费，巩固提升“双随机、一公开”监管成果。全面启动营商环境评价，营造便利高效的政务环境、开放公平的市场环境、公正透明的法治环境、诚实守信的信用环境。完成国企改革三年攻坚扫尾任务，持续完善产权结构、组织结构和治理结构，激发国企活力。落实支持民营企业政策，依法维护企业家人身权、财产权，构建“亲、清”新型政商关系，保护、培育、激励新时代企业家精神。加快农村综合改革，继续推进农村承包地三权分置，完成集体经济清产核资。深化“三地一矿”综合改革。完善高质量发展的指标体系、考核体系、评价体系。继续深化财税、水利、医疗、价格、科技、警务管理等领域改革。

    全面提升开放水平。大力实施新一轮高水平对外开放，以开放的主动赢得发展的主动、竞争的主动。积极融入“一带一路”，对接利用“四条丝路”，密切与长三角、珠三角、京津冀等发达地区的经济联系，打造全方位高层次大开放格局。加快建设安阳铁路口岸，积极申建安阳保税物流中心。狠抓资本招商、市场招商，强化现有企业对外合作招商。大力推进与上市公司、市场化产业基金、投资银行、资产公司合作，在教育、医疗、文化、制造业、现代农业、智慧城市、产业智能化改造、物流信息化提升等领域谋划包装一批项目，吸引外来投资。组织实施一批重大招商活动，全年引进省外资金、实际利用外资均增长3%左右。坚持诚信招商，政府出台的政策、作出的承诺，一定兑现到位。

    （五）统筹城乡，加快协调发展。深化国家文明城市创建，全面推进城市建设提质，大力实施乡村振兴战略，推动中心城区、县城和小城镇、农村一体化发展。

    推进城市高质量发展。坚持人民城市为人民，持续推动城市功能完善、品质提升、形象改善。强化规划引领。启动新一轮整体规划修编，推动多规合一，划定“三区三线”，优化城市布局，解决好制约城市发展的突出问题。完善专项规划，健全规划体系，严格规划执行。突出重点区域、重点片区、重点建筑，做好城市设计，落实功能定位，彰显城市特质。提升建设水平。继续实施园林绿化三年计划，着力打通“绿廊”、构筑“绿心”、加密“绿园”，新建公园游园等项目86个，新增绿地1.8万亩。加快洹河风光带和万金渠、邱家沟、示范区中轴线生态水系等项目实施，增加水量，改善水质，扩大水面，打造水景。提升主干道，打通断头路，增加路网密度，重点实施98项市政道路工程。继续推进老旧小区改造和背街小巷整治。加快公建配套类工程和“两场一所”建设。积极稳妥推进棚户区改造。加快古城保护整治复兴和示范街区建设，让一栋栋建筑、一座座设施、一片片区域把古都文脉彰显传承下去。加强城市管理。像绣花一样管理城市。深化城市综合执法改革，推动管理重心下移、执法力量下沉。整合城市管理信息系统，加强“城市大脑”建设。优先发展公共交通。加快推进生活垃圾分类。健全完善网格化管理制度，提升精细化管理水平。强力推进生态环境治污、交通秩序治堵、市容卫生治脏、公共服务治差。注重经营城市。强化土地收储利用，适度扩大土地供应规模，稳定土地市场。综合运用PPP模式、政策性金融和商业化运作等方式，引进战略投资者，集成包装供水、供气、供暖、垃圾处理、污水处理等有收益的项目，促进城市优质资产变现和公共服务提质提效。

    实施乡村振兴战略。牢牢把握“二十个字”总要求，保持历史耐心，科学规划、注重质量、从容建设，不断提升农业农村现代化水平。深化农业供给侧结构性改革，推进“四优四化”，促进农村一二三产业融合发展。全面落实粮食安全生产责任制，稳定粮食产能。全面完成“大棚房”专项清理。积极发展新型农业经营主体，强化新型农民培训，深化农村集体产权制度改革，提高农民工资性收入、财产性收入、经营性收入。持续改善农村人居环境，学习浙江“千村示范、万村整治”工程经验，统一规划、分步实施，整合资源、集中推进，全面抓好规划设计下乡入村，发挥好示范乡镇、示范村庄的引领带动作用，抓好农村“厕所革命”、污水垃圾处理等设施建设。深化文明村镇创建活动，推进乡风文明移风易俗，40%以上的行政村达到县级以上文明村标准。强化基层基础建设，创新乡村（社区）治理机制，提升治理能力和治理水平。

    大力发展县域经济。全面落实习近平总书记县域治理“三起来”、乡镇工作“三结合”的重要指示，推进县城提质发展和小城镇规范发展。综合考虑资源禀赋、产业定位、发展潜力，支持各县（市）因地制宜发展特色主导产业。强力推进百城建设提质，提高县城支撑带动作用，加快基础设施向农村延伸，推进基本公共服务标准化、均等化。选择一批中心镇，强化综合整治，提升公共服务水平，打造特色鲜明、管理规范、生态宜居的小城镇。

    （六）夯实基础，提升支撑能力。加快布局和实施一批打基础利长远的基础设施项目，增创综合发展优势，更好支撑高质量发展。

    强化交通支撑。实施“公转铁”行动，建设一批连通大型工业企业、物流园区的铁路专用线，推进安李铁路电气化改造，确保象道物流、万庄物流铁路专用线投用。加快推进安阳机场建设。力争开工建设高铁客运枢纽站、沿太行高速公路安阳段等重大项目。积极推进安林市域铁路、安罗高速公路安阳段前期工作。按期推进新S301等25个公路项目，建成安内快速通道。实施一批多式联运示范工程，打造内畅外捷、通达四方的综合交通体系。

    强化水利支撑。坚持水资源、水生态、水环境、水灾害“四水同治”，实施一批引水、治水、管水工程。推进城市水系连通，加强河湖岸线管理，抓好病险水闸水库除险加固，做好洹河综合治理、宝莲湖南水北调调蓄工程前期，确保引岳入安二期建成通水。

    强化能源支撑。优化能源供给结构，大力发展清洁能源。继续实施风电光电“双百万工程”，重点抓好大唐滑县枣村300兆瓦、华润安阳县永和250兆瓦等风电项目。积极争取扩大天然气供应规模。加快推进京煤滑州热电联产、汤阴华能背压机组项目建设，做好内黄县、文峰区地热能清洁供暖规模化利用试点工作。加强城乡配电网建设，提升农村电网保障能力。

    强化生态支撑。实施国土绿化提速行动，推进山区森林化、平原林网化、城市园林化、乡村林果化、廊道林荫化、庭院花园化。加强国储林建设，实现“双环六廊”城市防护林闭合，营造林34.8万亩，森林抚育改造12万亩，建成国家森林城市。加大自然保护区管理保护力度，确保漳河峡谷、淇淅河两个国家湿地公园通过国家验收。推动南太行“山水林田湖草”综合治理试点建设，持续推进西部矿区生态修复。

    强化网络支撑。积极完善新一代信息基础设施，争取5G商用试点布局，大力引进培育关键支撑技术企业、数据增值服务企业、终端产品制造企业，推进高新区大数据产业园建设。加快“智慧视频云”“智能交通”建设。

    （七）改善民生，推动共享发展。坚持把民生改善作为发展的出发点和落脚点，持续优化公共服务供给，坚决办好群众关心的民生实事，多谋民生之利，多解民生之忧。

    着力稳就业促创业。全方位优化公共就业服务，推进全民技能振兴工程，做好高校毕业生、退役军人、“僵尸企业”处置和化解过剩产能分流职工等重点群体就业。扶持灵活就业和新就业形态。加强城镇困难人员就业援助。鼓励支持农民工返乡创业。完成职业培训3.5万人次。新增农村劳动力转移就业1.8万人。

    提升社会保障水平。深入实施全民参保计划。继续提高城乡居民基础养老金最低标准，继续调增企业和机关事业单位退休人员基本养老金。持续提升基本医保、大病医保、困难群众大病补充医保保障水平。进一步提高城乡低保、特困供养、孤儿养育等保障标准。实施残疾儿童康复救助。加强残疾人权益保障。做好临时救助工作。

    坚持教育优先发展。推动城乡义务教育一体化发展，新建改扩建中小学13所，持续解决大班额问题，减轻中小学生课外负担，规范校外培训机构。加快实施第三期学前教育行动计划。大力引进名校来安合作办学，促进高中教育多样化特色化发展、高等教育内涵发展。支持职业院校开展校企合作、建设实训基地。做好民办教育分类管理。全年新增学位27000个以上。加强教师队伍建设。深入开展学校及周边环境综合治理，切实维护校园安全。

    推进健康安阳建设。加快区域医疗中心建设，提升基层医疗机构标准化水平，完善公共卫生服务体系。实施健康素养促进行动。深化综合医改，积极组建城市医疗集团和县域医共体。加快医保支付方式改革，提高医保资金使用效益。探索发展“互联网+医疗健康”。大力支持社会办医。广泛开展全民健身活动，办好第十二届市运会。积极创建国家食品安全示范城市，严格食品药品安全监管，坚决把好人民群众饮食用药安全每一道关口。

    繁荣发展文化事业。积极践行社会主义核心价值观，大力弘扬红旗渠精神。广泛开展群众性精神文明创建。推动公共文化服务体系提质增效，加快推进一批重点文化项目建设，做实文化下基层、文化惠民活动。支持创作文艺精品。持续推进“扫黄打非”，净化文化市场。以殷墟遗址保护为带动，全面推行文物考古勘探前置工作，提升全市文物保护水平，做好文物活化利用工作。积极推动红旗渠申遗。办好甲骨文发现120周年系列纪念活动。传承创新地域特色文化，保护好千年古都的根与魂。

    提升社会治理水平。学习“枫桥经验”，全面加强基层社会治理。依法做好信访工作。深入开展普法教育，做好法治宣传“六进”。加强社会治安防控体系建设，推进“一村（格）一警”工作。深化扫黑除恶专项斗争。扎实做好庆祝新中国成立70周年安保等工作，维护社会大局和谐稳定。

    支持国防和军队改革，抓好民兵和人民防空建设，深入开展“双拥”共建，完善退役军人服务保障体系，巩固军政、军民团结。全面落实党的民族政策和宗教工作基本方针，促进民族团结、宗教和睦。加强老区建设。支持工会、共青团、妇联等人民团体更好发挥桥梁纽带作用。做好老龄和关心下一代工作。高质量完成第四次经济普查。加强史志、档案、气象、地震、邮政、外事侨务、援疆等工作。

    继续办好重点民生实事：一是提高高龄老人医保报销比例；二是新建改扩建公办幼儿园39所；三是开展妇女“两癌”免费筛查，开展居民（50—59岁）消化道肿瘤和心脑血管病免费筛查；四是建设全民健身体育设施185处；五是新建改建城区标准化农贸市场5个；六是新增公共停车位10000个，新增充电桩500个；七是建设城市书屋6座；八是开工建设南水北调西部供水工程；九是新改建农村公路150公里以上；十是新增100个行政村通公交。

    三、加强政府自身建设

    新时代新使命对政府工作提出了新要求。我们将进一步提高政治站位，强化使命担当，加快职能转变，提升治理能力，努力建设人民满意的服务型政府。

    （一）加强政治建设。坚持以习近平新时代中国特色社会主义思想武装头脑、指导实践、推动工作，树牢“四个意识”，坚定“四个自信”，坚决维护习近平总书记党中央的核心、全党的核心地位，坚决维护党中央权威和集中统一领导，做到有令则行、有禁必止，决不允许不敬畏、不在乎、喊口号、装样子。坚持把党对一切工作的领导贯彻到政府工作的各领域各方面，确保习近平总书记的指示批示精神、党中央的大政方针和省委、市委决策部署落地见效。坚持从政治角度看待问题，用政治责任推动工作，以政治效果评判工作，把讲政治体现在岗位上、落实到行动中。

    （二）坚持依法行政。弘扬法治精神，以法治思维和法治方式用好“有形之手”、管住“妄动之举”，做到“法无授权不可为，法定职责必须为”。坚持依法决策，加强重大行政决策、规范性文件、重大行政执法决定合法性审查，凡是涉及重大公共利益、群众切身利益的事项，都要广泛征求各方面意见，切实提高政府决策科学化、民主化、法治化水平。自觉接受人大监督、政协监督和监察监督，加强审计监督，主动接受社会和舆论监督，让透明成为常态，让权力在阳光下运行。

    （三）强化务实担当。政府工作的关键是落实，推进落实的根本在担当。要始终保持永不懈怠的精神状态和一往无前的奋斗姿态，积极投身改革发展的主战场、攻坚克难的第一线、服务群众的最前沿，以对群众的亲劲、抓工作的韧劲、干事业的拼劲，在担责担难担险中体现作为、彰显价值。对定下来的事情雷厉风行、抓紧实施，对部署的工作跟踪问效、一抓到底，以实干实效取信于民。锤炼“五个过硬”，增强“八种本领”，大力倡树“五比五不比”，全面加强政府系统干部队伍建设，使学的氛围、严的氛围、干的氛围越来越浓。坚持严管与厚爱结合、激励和约束并重，认真落实“三个区分开来”，为担当者担当，为负责者负责，为干事者撑腰，努力营造“以担当为荣、以发展为重、以奋斗为乐”的良好局面。

    （四）践行为民宗旨。离开人民，我们寸步难行；依靠人民，我们无往不胜。政府工作人员要坚持走好新形势下的群众路线，思想上尊重群众、作风上联系群众、工作上为了群众，始终与群众站在一起、想在一起、干在一起，紧盯群众心中的痛点、难点、堵点，实打实解决群众最盼、最忧、最急、最怨的切身利益问题，让全市人民生活更方便、更舒心、更美好，让我们的城市更有温度。

    （五）恪守清正廉洁。坚持把纪律和规矩挺在前面，以“永远在路上”的执着，全面加强清廉政府建设。严格落实中央八项规定及其实施细则精神，严防“四风”问题反弹回潮，严肃整治形式主义、官僚主义新表现。严格遵守廉洁自律准则，筑牢思想防线，保持清醒头脑，始终敬畏人民、敬畏组织、敬畏法纪，在各式各样的陷阱和形形色色的围猎面前，自觉划清边线、不碰红线、守住底线。坚决落实全面从严治党各项要求，切实做好各类巡视巡察督查反馈问题整改，坚决防止和纠正损害群众利益的不正之风。加强党风廉政教育，推进廉政文化建设，让铁规铁纪成为政府工作人员的自觉遵循。

    各位代表！

    百舸争流奋楫者先，千帆竞发勇进者胜。让我们更加紧密地团结在以习近平同志为核心的党中央周围，在市委的坚强领导下，忠诚履职尽责，主动担当作为，开拓创新，砥砺前行，奋力谱写重返全省第一方阵、决胜全面小康、建设新时代区域性中心强市新篇章，以优异成绩迎接新中国成立70周年！
